# Soft Substructures in Quantales and Their Approximations Based on Soft Relations

**DOI:** 10.1155/2022/6820719

**Published:** 2022-08-02

**Authors:** Huan Zhou, Saqib Mazher Qurashi, Muti Ur Rehman, Muhammad Shabir, Rani Sumaira Kanwal, Ahmed Mostafa Khalil

**Affiliations:** ^1^Aviation Engineering School, Air Force Engineering University, Xi'an 710038, China; ^2^Government College University Faisalabad, Faisalabad, Pakistan; ^3^Quaid-i-Azam University Islamabad, Islamabad, Pakistan; ^4^Department of Mathematics, Faculty of Science, Al-Azhar University, Assiut 71524, Egypt

## Abstract

The aim of this research article is to derive a new relation between rough sets and soft sets with an algebraic structure quantale by using soft binary relations. The aftersets and foresets are utilized to define lower approximation and upper approximation of soft subsets of quantales. As a consequence of this new relation, different characterization of rough soft substructures of quantales is obtained. To emphasize and make a clear understanding, soft compatible and soft complete relations are focused, and these are interpreted by aftersets and foresets. Particularly, in our work, soft compatible and soft complete relations play an important role. Moreover, this concept generalizes the concept of rough soft substructures of other structures. Furthermore, the algebraic relations between the upper (lower) approximation of soft substructures of quantales and the upper (lower) approximation of their homomorphic images with the help of soft quantales homomorphism are examined. In comparison with the different type of approximations in different type of algebraic structures, it is concluded that this new study is much better.

## 1. Introduction

Quantale theory was proposed by Mulvey[1]. It is based on defining an algebraic structure on a complete lattice. Since quantale was defined on a complete lattice, there must be a correlation between linear logic and quantale theory which was studied by Yetter, in his study. He presented a new class of models for linear intuitionistic logic [2]. In recent years, quantale is applied in vast research areas, such as algebraic theory [3], rough set theory [4–7], topological theory [8], theoretical computer science [9], and linear logic [10].

In 1982, Pawlak developed the famous rough set theory [11], which is a mathematization of inadequate knowledge. The rough set deals with the categorization and investigation of inadequate information and knowledge. After Pawlak's work, Zhu [12] provided some new views on the rough set theory. In [13], Ali et al. studied some properties of generalized rough sets. Nowadays, rough sets are applied in many different areas, such as cognitive sciences, machine learning, pattern recognition, and process control.

There are many problems that arise in different fields such as engineering, economics, and social sciences in which data have some sort of uncertainty. Well-known mathematical tools have so many limitations because these tools are introduced for particular circumstances. There are many theories to overcome uncertainty such as fuzzy set theory, probability theory, rough sets, and vague sets, but these are limited due to its design.

In 1998, Molodtsov present the idea of soft set theory, which is a mathematical tool to overcome the adversities affecting the above theories [14]. Many authors like Maji et al. present different operations on soft sets and try to consolidate the algebraic aspects of soft sets [15]. A new and different idea of operations was presented by Ali et al. [16]. Many soft algebraic structures such as soft modules [17], soft groups [18], soft rings [19], and soft ordered semigroups [20] were studied. The basic theme and purpose of soft sets are to create the idea of parametrization, and this idea has been utilized to find soft binary relation (SBR) which is a parameterized collection of binary relations on a universe under consideration. This puts forward the consideration for complicated objects that may be perceived from different points of view. In [21–23], Feng et al. presented the relationship between soft, rough, and fuzzy sets and produced rough soft sets, soft rough sets, and soft-rough fuzzy sets.

By using aftersets and foresets notions associated with SBR, a new approximation space is widely utilized these days. By using generalized approximation space based on SBR, different soft substructures in semigroups were approximated by Kanawal and Shabir [24]. Motivated by the idea in [24], soft substructures in quantales are defined, and the aftersets and foresets are employed to construct the lower approximation and upper approximation of soft substructures. Since we are dealing with the approximation of soft subsets of quantale, further soft substructures are employed for further characterization.

There are several authors who introduced rough sets theory in algebraic structures and soft algebraic structures. Iwinski analyzes algebraic properties of rough sets [25]. Qurashi and Shabir present the idea of roughness in *Q*- module [5]. Idea of the generalized rough quantales (subquantales) was presented by Xiao and Li [6]. Rough prime (semiprime and primary) ideals in quantales were investigated by Yang and Xu [7]. Fuzzy ideals (prime, semiprime, and primary) in quantales were introduced by Luo and Wang [4]. Generalized roughness of fuzzy substructures in quantale is studied by Qurashi et al. [26]. In [27], Yamak et al. proposed the idea of set-valued mappings as the basis of the generalized upper (lower) approximations of a ring with the help of ideals. Rough prime bi Γ-hyper ideals of Γ-semihypergroups were proposed by Yaqoob et al. [28, 29]. Rough substructures of semigroups were studied by Kuroki [30].

The following scheme is designed for the rest of the paper. Some essential explanations related to quantales, its substructures, soft substructures, and their corresponding sequels are connected in Section 2. Notion of approximations of soft sets over quantale generated by soft binary relations is discussed in Section 3. In Section 4, by using these ideas, generalized soft substructures are defined and investigated further fundamental algebraic characteristics of these phenomena. Additionally, we extend this study to define the relationship between homomorphic images and their approximation by soft binary relation in Section 5.

## 2. Preliminaries

Let Θ be a nonempty finite set called the universe set and Ψ be an E.R (equivalence relation) over Θ. Let [*q*]_Ψ_ denotes the equivalence class of the relation containing *q*. Any definable set in Θ would be written as finite union of equivalence classes of Θ. Let *R*⊆Θ in general *R* is not a definable set in Θ. However, the set *R* can be approximated by two definable sets in Θ. The first one is called Ψ-lower approximation (Ψ − L_appr_) of *R*, and the second is called Ψ-upper approximation (Ψ − *U*_appr_). They are defined as follows:

(1) $$\begin{array}{c} \underline{\Psi }\left(R\right)=\left\{q\in \Theta :{\left[q\right]}_{\Psi }\subseteq R\right\}. \end{array}$$

(2) $$\begin{array}{c} \bar{\Psi }\left(R\right)=\left\{q\in \Theta :{\left[q\right]}_{\Psi }\cap R\neq \emptyset \right\}. \end{array}$$

The Ψ − *L*_appr_ of *R* in Θ is the greatest definable in Θ contained in *R*. The Ψ − *U*_appr_ of *R* in Θ is the least definable set in Θ containing *R*. For any nonempty subset *R* in Θ, $\Psi \left(R\right)=\left(\underline{\Psi }\left(R\right),\bar{\Psi }R\right)$ is called rough set with respect to Ψ or simply a Ψ-rough subset of *P*(Θ) × *P*(Θ) if $\underline{\Psi }\left(R\right)\neq \bar{\Psi }\left(R\right)$, where *P*(Θ) denotes the set of all subsets of Θ.

**Definition 1 (see [31]).**

Let Θ be a complete lattice. Define an associative binary relation ∘ on Θ satisfying

(3) $$\begin{array}{c} l\circ \left(\vee_{i\in I}w_{i}\right)=\vee_{i\in I}\left(l\circ w_{i}\right)\text{ and }\left(\vee_{i\in I}l_{i}\right)\circ w=\vee_{i\in I}\left(l_{i}\circ w\right), \end{array}$$

∀ *l*,  *w*,  *l*_*i*_,  *w*_*i*_ ∈ Θ. Then, (Θ, ∘) is called quantale.

Let *T*_1_,  *T*_2_,  *T*_*I*_ ⊂ Θ, *i* ∈ *I*. We define some notions as follows:

(4) $$\begin{array}{c} T_{1}{}^{\circ}T_{2}=\left\{t_{1}{}^{\circ}t_{2}:t_{1}\in T_{1},t_{2}\in T_{2}\right\};\\ T_{1}\vee T_{2}=\left\{t_{1}\vee t_{2}:t_{1}\in T_{1},t_{2}\in T_{2}\right\};\\ \vee_{i\in I}T_{i}=\left\{\vee_{i\in I}t_{i}:t_{i}\in T_{i}\right\}. \end{array}$$

Throughout the paper, quantales are denoted by Θ_1_ and Θ_2_.

Let ∅≠*W*⊆Θ. Then, _*W*_ is called a subquantale of Θ if the following holds:

1. *w*_1_°*w*_2_ ∈ *W*, ∀*w*_1_, *w*_2_ ∈ *W*.
2. ∨_*i*∈*I*_*w*_*i*_, ∈*W*, ∀*w*_*i*_, ∈*W*.

That is, Θ closed under ∘ and arbitrary supremum.

**Definition 2 (see [32]).**

Let Θ be a quantale, ∅≠*I*⊆Θ is called left (right) ideal if the following satisfied:

1. *u*,  *v* ∈ *I* implies *u*∨*v* ∈ 1
2. *p* ∈ Θ, *u* ∈ *I* such that *p* ≤ *u* implies *p* ∈ *I*
3. *q* ∈ Θ and *u* ∈ *I* implies *q*°*u* ∈ *I*(*u*°*q* ∈ *I*)

A nonempty subset *I*⊆Θ is called ideal of Θ if it is left as well as right ideal.

**Example 1 .**

**Figure 1:**
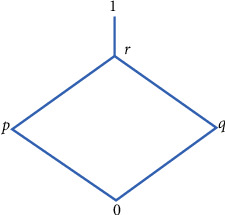
Illustration of Θ.

Let Θ*=*{0,  *p*,  *q*,  *r*,  1} complete lattices are shown in Figure 1. We define ∘ be the associative binary operation on Θ as shown in Table 1.

Then, Θ is a quantale. Then, {0}, {0, *p*}, {0, *q*}, {0,  *p*, *q*,  *r*}, and Θ are all *I* of quantale Θ.

**Definition 3 (see [32]).**

Let ∅≠*I*⊆Θ be an ideal. *I* is called prime ideal if, ∀*u*,  *v* ∈ Θ, *u*°*v* ∈ *I*⇒*u* ∈ *I* or *v* ∈ *I*. *I* is called semiprime *I* if, ∀ *u* ∈ Θ, *u*°*u* ∈ *I*⇒*u* ∈ *II* is called primary *I* if, ∀*u*,  *v* ∈ Θ, *u*°*v* ∈ *I* and *u* ∉ *I* implies *v*^*n*^ ∈ *I* for some *n* ∈ *Ν*.

**Definition 4 (see [14]).**

A pair (Ψ, *C*) is called a soft set over Θ if Ψ : *C*⟶*P*(Θ) where *C* is a subset of *E* (the set of parameters).

**Definition 5 (see [16]).**

Let (*F*, *C*_1_) and (*H*, *C*_2_) be two soft sets over Θ. Then, (*F*, *C*_1_) soft subset (*H*, *C*_2_) if the following conditions are fulfilled:

1. *C*_1_⊆*C*_2_
2. *F*(*c*)⊆*H*(*c*), ∀ c ∈ *C*_1_

**Definition 6 (see [33]).**

Let (Ψ, *C*) be a soft set over Θ × Θ, that is, Ψ : *C*⟶*P*(Θ × Θ). Then, (Ψ, *C*) is called a soft binary relation (SBR) over Θ × Θ. A SBR over Θ_1_ × Θ_2_ is a soft set (Ψ, *C*) over Θ_1_ × Θ_2_. That is, Ψ : *C*⟶*P*(Θ_1_ × Θ_2_).

**Definition 7 .**

Let (Ψ, *C*) be a soft set over quantale Θ. Then,

1. (Ψ, *C*) is called soft subquantale over Θ iff Ψ(*c*) is a subquantale of Θ, ∀ c ∈ *C*
2. (Ψ, *C*) is called soft ideal over Θ iff Ψ(*c*) is an ideal of Θ, ∀ c ∈ *C*
3. (Ψ, *C*) is called soft prime ideal over Θ iff Ψ(*c*) is a prime ideal of Θ, ∀ c ∈ *C*
4. (Ψ, *C*) is called soft semiprime ideal over Θ iff Ψ(*c*) is a semiprime ideal of Θ, ∀ c ∈ *C*
5. (Ψ, *C*) is called soft primary ideal over Θ iff Ψ(*c*) is a primary ideal of Θ, ∀ c ∈ *C*

## 3. Approximation of Soft Sets over Quantale by Soft Binary Relation

In this section, we present some important aspects regarding to the approximation of soft sets in quantale Θ by SBR. We utilized aftersets and foresets to approximate soft sets.

**Definition 8 (see [34]).**

Let (Ψ, *C*) be a SBR over Θ_1_ × Θ_2_, where *C*⊆*E* (parametric set). Then, Ψ : *C*⟶*P*(Θ_1_ × Θ_2_). For a soft set (*F*, *C*) over Θ_2_, the *L*_appr_$\left({\underline{\Psi }}^{F},C\right)$ and *U*_appr_$\left({\bar{\Psi }}^{F},C\right)$ of (*F*, *C*) w.r.t the afterset are essentially two soft sets over Θ_1_, which is defined as

(5) $$\begin{array}{c} {\underline{\Psi }}^{F}\left(c\right)=\left\{q_{1}\in \Theta_{1}:\emptyset \neq q_{1}\Psi \left(c\right)\subseteq F\left(c\right)\right\}, \end{array}$$

(6) $$\begin{array}{c} {\bar{\Psi }}^{F}\left(c\right)=\left\{q_{1}\in \Theta_{1}:q_{1}\Psi \left(c\right)\cap F\left(c\right)\neq \emptyset \right\},\;\forall ,\;c\in C. \end{array}$$

And for a soft set (*H*, *C*) over Θ_1_, the *L*_appr_Ψ¯H,C and *U*_appr_Ψ¯H,C of (*H*, *C*) w.r.t the foreset are actually two soft sets over Θ_2_, which is defined as

(7) Ψ¯Hc=q2∈Θ2:∅≠Ψcq2⊆Hc,Ψ¯Hc=q2∈Θ2:Ψcq2∩Hc≠∅.

For all c ∈ *C*, where *q*_1_Ψ(*c*)*=*{*q*_2_ ∈ Θ_2_ : (*q*_1_, *q*_2_) ∈ Ψ(*c*)} is called the afterset of *q*_1_ and Ψ(*c*)*q*_2_*=*{*q*_1_ ∈ Θ_1_ : (*q*_1_, *q*_2_) ∈ Ψ(*c*)} is called the foreset of *q*_2_.

**Remark 1 .**

1. For each soft set (*F*, *C*) over Θ_2_, ${\underline{\Psi }}^{F}:C⟶P\left(\Theta_{1}\right)$ and ${\bar{\Psi }}^{F}:C⟶P\left(\Theta_{1}\right)$
2. For each soft set (*H*, *C*) over Θ_1_, Ψ¯H:C⟶PΘ2 and Ψ¯H:C⟶PΘ2

**Definition 9 .**

Let (Ψ, *C*) be a SBR over Θ_1_ × Θ_2_, that is, Ψ : *C*⟶*P*(Θ_1_ × Θ_2_). Then, (Ψ, *C*) is called soft compatible relation (SCPR) if for all *p*,  *r*,  *j*_*i*_ ∈ Θ_1_ and *q*,  *s*,  *k*_*i*_ ∈ Θ_2_(*i* ∈ *I*), we have

1. (*p*, *q*), (*r*, *s*) ∈ Ψ(*c*)⇒(*p*∘_1_*r*, *q*∘_2_*s*)∈Ψ(*c*)
2. (*j*_*i*_,  k_*i*_) ∈ Ψ(*c*)⇒(∨_*i*∈*I*_*j*_*i*_, ∨_*i*∈*I*_*k*_*i*_)∈Ψ(*c*)

for every c ∈ *C*.

**Definition 10 .**

A SCPR (Ψ, *C*) over Θ_1_ × Θ_2_ is called soft complete relation (SCTR) with respect to the afterset if, for all *p*,  *r*,   ∈ Θ_1_, we have

1. *p*Ψ(*c*)∨*r*Ψ(*c*)*=*(*p*∨*r*)Ψ(*c*)
2. *p*Ψ(*c*)∘_2_*r*Ψ(*c*)*=*(*p*∘_1_*r*)Ψ(*c*)

for all *c* ∈ *C*.

A SCPR (Ψ, *C*) is called ∨-complete w.r.t the aftersets if it satisfies only condition (1). A SCPR (Ψ, *C*) is called °-complete w.r.t the aftersets if it satisfies only condition (2).

A SCPR (Λ, *C*) over Θ_1_ × Θ_2_ is called soft complete relation (SCTR) with respect to the foreset if for all *q*,  *s* ∈ Θ_2_, and we have

1. Λ(*c*)*q*∨Λ(*c*)*s=*Λ(*c*)(*q*∨*s*)
2. Λ(*c*)*q*∘_1_Λ(*c*)*s=*Λ(*c*)(*q*∘_2_*s*)

for all *c* ∈ *C*.

A SCPR (Λ, *C*) is called ∨-complete w.r.t the foresets if it satisfies only condition (1).

A SCPR (Λ, *C*) is called °-complete w.r.t the foresets if it satisfies only condition (2).

**Theorem 1 .**

Let (Ψ, *C*) be a SCPR with respect to the afterset over Θ_1_ × Θ_2_. Then, for any two soft sets (*F*_1_, *C*) and (*F*_2_, *C*) over Θ_2_, we have

1. $\left({\bar{\Psi }}^{F_{1}},C\right)\circ_{1}\left({\bar{\Psi }}^{F_{2}},C\right)\subseteq \left({\bar{\Psi }}^{F_{1}\circ_{2}F_{2}},C\right)$
2. $\left({\bar{\Psi }}^{F_{1}},C\right)\vee \left({\bar{\Psi }}^{F_{2}},C\right)\subseteq \left({\bar{\Psi }}^{F_{1}\vee F_{2}},C\right)$

**Proof**

For arbitrary *c* ∈ *C*, let $x\in {\bar{\Psi }}^{F_{1}}\left(c\right)\circ_{1}{\bar{\Psi }}^{F_{2}}\left(c\right).$ Then, *x=y*_1_∘_1_*y*_2_ for some $y_{1}\in {\bar{\Psi }}^{F_{1}}\left(c\right)$ and $y_{2}\in {\bar{\Psi }}^{F_{2}}\left(c\right)$. This implies that *y*_1_Ψ(*c*)∩*F*_1_(*c*) ≠ ∅ and *y*_2_Ψ(*c*)∩*F*_2_(*c*) ≠ ∅, so there exist elements *l*,  *m* ∈ Θ_2_ such that *l* ∈ *y*_1_Ψ(*c*)∩*F*_1_(*c*) and *m* ∈ *y*_2_Ψ(*c*)∩*F*_2_(*c*). Thus, *l* ∈ *y*_1_Ψ(*c*),  *m* ∈ *y*_2_Ψ(*c*),  *l* ∈ *F*_1_(*c*) and *m* ∈ *F*_2_(*c*). So (*y*_1_, *l*) ∈ Ψ(*c*) and (*y*_2_, *m*) ∈ Ψ(*c*) imply (*y*_1_∘_1_*y*_2_, *l*∘_2_*m*) ∈ Ψ(*c*); that is, (*l*∘_2_*m*) ∈ (*y*_1_∘_1_*y*_2_)Ψ(*c*). Also, *l*∘_2_*m*∈*F*_1_(*c*)∘_2_*F*_2_(*c*); therefore, *l*∘_2_*m* ∈ *y*_1_∘_1_*y*_2_Ψ(*c*)∩*F*_1_(*c*)∘_2_*F*_2_(*c*). This shows that $x=y_{1}\circ_{1}y_{2}\in {\bar{\Psi }}^{F_{1}\circ_{2}F_{2}}\left(c\right).$

Now, for arbitrary *c* ∈ *C*, let $x\in {\bar{\Psi }}^{F_{1}}\left(c\right)\vee {\bar{\Psi }}^{F_{2}}\left(c\right).$ Then, *x=y*_1_∨*y*_2_ for some $y_{1}\in {\bar{\Psi }}^{F_{1}}\left(c\right)$ and $y_{2}\in {\bar{\Psi }}^{F_{2}}\left(c\right)$. This implies that *y*_1_Ψ(*c*)∩*F*_1_(*c*) ≠ ∅ and *y*_2_Ψ(*c*)∩*F*_2_(*c*) ≠ ∅, so there exist elements *l*,  *m* ∈ Θ_2_ such that *l* ∈ *y*_1_Ψ(*c*)∩*F*_1_(*c*) and *m* ∈ *y*_2_Ψ(*c*)∩*F*_2_(*c*). Thus, *l* ∈ *y*_1_Ψ(*c*),  *m* ∈ *y*_2_Ψ(*c*),  *l* ∈ *F*_1_(*c*), and *m* ∈ *F*_2_(*c*). So (*y*_1_, *l*) ∈ Ψ(*c*) and (*y*_2_, *m*) ∈ Ψ(*c*) imply (*y*_1_∨*y*_2_, *l*∨*m*) ∈ Ψ(*c*); that is, (*l*∨*m*) ∈ (*y*_1_∨*y*_2_)Ψ(*c*). Also, *l*∨*m* ∈ *F*_1_(*c*)∨*F*_2_(*c*); therefore, *l*∨*m* ∈ *y*_1_∨*y*_2_Ψ(*c*)∩*F*_1_(*c*)∨*F*_2_(*c*). This shows that $x=y_{1}\vee y_{2}\in {\bar{\Psi }}^{F_{1}\vee F_{2}}\left(c\right).$

**Theorem 2 .**

Let (Ψ, *C*) be a SCPR with respect to the foreset over Θ_1_ × Θ_2_. Then, for any two soft sets (*L*_1_, *C*) and (*L*_2_, *C*) over Θ_1_, we have

1. Ψ¯L1,C∘2Ψ¯L2,C⊆Ψ¯L1∘1L2,C
2. Ψ¯L1,C∨Ψ¯L2,C⊆Ψ¯L1∨L2,C

**Proof**

The proof is simple.

**Theorem 3 .**

Let (Ψ, *C*) be a SCTR w.r.t the afterset over Θ_1_ × Θ_2_. Then, for any two soft sets (*F*_1_, *C*) and (*F*_2_, *C*) over Θ_2_, we have

1. $\left({\underline{\Psi }}^{F_{1}},C\right)\circ_{1}\left({\underline{\Psi }}^{F_{2}},C\right)\subseteq \left({\underline{\Psi }}^{F_{1}\circ_{2}F_{2}},C\right)$
2. $\left({\underline{\Psi }}^{F_{1}},C\right)\vee \left({\underline{\Psi }}^{F_{2}},C\right)\subseteq \left({\underline{\Psi }}^{F_{1}\vee F_{2}},C\right)$

**Proof**

For arbitrary *c* ∈ *C*, if at least one of Ψ^*F*_1_^(*c*) and Ψ^*F*_2_^(*c*) is empty, then (1) is obvious. Now, for arbitrary *c* ∈ *C*, consider that Ψ^*F*_1_^(*c*) ≠ ∅ and Ψ^*F*_2_^(*c*) ≠ ∅. Then, Ψ^*F*_1_^(*c*)°_1_Ψ^*F*_2_^(*c*) ≠ ∅. So, let *x* ∈ Ψ^*F*_1_^(*c*)°_1_Ψ^*F*_2_^(*c*). Then, *x=y*_1_°_1_*y*_2_ for some *y*_1_ ∈ Ψ^*F*_1_^(*c*) and *y*_2_ ∈ Ψ^*F*_2_^(*c*). This implies that ∅≠*y*_1_Ψ(*c*)⊆*F*_1_(*c*) and ∅≠*y*_2_Ψ(*c*)⊆*F*_2_(*c*). As (*y*_1_°_1_*y*_2_)Ψ(*c*)*=y*_1_Ψ(*c*)°_2_Ψ(*c*)⊆*F*_1_(*c*)°_2_*F*_2_(*c*). This shows that *x=y*_1_°_1_*y*_2_ ∈ Ψ^*F*_1_*F*_2_^(*c*). Hence, (1) is proved.

For arbitrary *c* ∈ *C*, if at least one of Ψ^*F*_1_^(*c*) and Ψ^*F*_2_^(*c*) is empty, then (2) is obvious. Now, for arbitrary *c* ∈ *C*, consider that Ψ^*F*_1_^(*c*) ≠ ∅ and Ψ^*F*_2_^(*c*) ≠ ∅. Then, Ψ^*F*_1_^(*c*)∨Ψ^*F*_2_^(*c*) ≠ ∅. So, let *x* ∈ Ψ^*F*_1_^(*c*)∨Ψ^*F*_2_^(*c*). Then, *x=y*_1_∨*y*_2_ for some *y*_1_ ∈ Ψ^*F*_1_^(*c*) and *y*_2_ ∈ Ψ^*F*_2_^(*c*). This implies that ∅≠*y*_1_Ψ(*c*)⊆*F*_1_(*c*) and ∅≠*y*_2_Ψ(*c*)⊆*F*_2_(*c*). As (*y*_1_∨*y*_2_)Ψ(*c*)*=y*_1_Ψ(*c*)∨*y*_2_Ψ(*c*)⊆*F*_1_(*c*)∨*F*_2_(*c*). This shows that *x=y*_1_∨*y*_2_ ∈ Ψ^*F*_1∨*F*2_^(*c*). Hence, (2) is proved.

**Theorem 4 .**

Let (Ψ, *C*) be a SCTR with respect to the foreset over Θ_1_ × Θ_2_. Then, for any two soft sets (*L*_1_, *C*) and (*L*_2_, *C*) over Θ_1_, we have

1. Ψ¯L1,C∘2Ψ¯L2,C⊆Ψ¯L1∘1L2,C
2. Ψ¯L1,C∨Ψ¯L2,C⊆Ψ¯L1∨L2,C

**Proof**

The proof is obvious.

## 4. Approximation of Soft Substructures in Quantales

In this section, we consider two quantales Θ_1_ and Θ_2_ and approximate different soft substructures of quantales by using different SBR over Θ_1_ × Θ_2_. We will show that *U*_appr_ of a soft substructure of quantales by using SCPR is again a soft substructure of quantales and provide counter examples to support the argument that the converse is not true. Also, we will show that *L*_appr_ of a soft substructure of quantales by using SCTR is again a soft substructure of quantales and provide a counter example to support the argument that the converse is not true.

Throughout this section, we consider (Ψ, *C*) to be the SBR over Θ_1_ × Θ_2_ and *x*Ψ(*c*) ≠ ∅ for all *x* ∈ Θ_1_, *c* ∈ *C*, and Ψ(*c*)*y* ≠ ∅ for all *y* ∈ Θ_2_, *c* ∈ *C* unless otherwise specified.

**Definition 11 .**

Let (Ψ, *C*) be a SBR over Θ_1_ × Θ_2_ and (*F*, *C*) be a soft set over Θ_2_. If *U*_appr_. $\left({\bar{\Psi }}^{F},C\right)$ is a soft subquantale of Θ_1_, then (*F*, *C*) is called generalized upper soft (*GU*_*p*_*S*) subquantale of Θ_1_ w.r.t the aftersets. If *U*_appr_$\left({\bar{\Psi }}^{F},C\right)$ is a soft ideal (prime ideal, semiprime ideal, and primary ideal) of Θ_1_, then (*F*, *C*) is called *GU*_*p*_*S* ideal (prime ideal, semiprime ideal, and primary ideal) of Θ_1_ w.r.t the aftersets.

**Definition 12 .**

Let (Ψ, *C*) be a SBR over Θ_1_ × Θ_2_ and (*L*, *C*) be a soft set over Θ_1_. If *U*_appr_Ψ¯L,C is a soft subquantale of Θ_2_, then (*L*, *C*) is called generalized upper soft (*GU*_*p*_*S*) subquantale of Θ_2_ w.r.t the foresets. If *U*_appr_Ψ¯L,C is a soft ideal (prime ideal, semiprime ideal, and primary ideal) of Θ_2_, then (*L*, *C*) is called *GU*_*p*_*S* ideal (prime ideal, semiprime ideal, and primary ideal) of Θ_2_ w.r.t the foresets.

**Theorem 5 .**

Let (Ψ, *C*) be a SCPR over Θ_1_ × Θ_2_. If (*F*, *C*) is a soft subquantale of Θ_2_, then (*F*, *C*) is a *GU*_*p*_*S* subquantale of Θ_1_ w.r.t the aftersets.

**Proof**

Suppose that (*F*, *C*) is a soft subquantale, then $\emptyset \neq {\bar{\Psi }}^{F}\left(c\right)$ for any *c* ∈ *C*. Let $p_{i}\in {\bar{\Psi }}^{F}\left(c\right)$, *i* ∈ *I*. Then, *p*_*i*_Ψ(*c*)∩*F*(*c*) ≠ ∅. So, there exists *q*_*i*_ ∈ *p*_*i*_Ψ(*c*)∩*F*(*c*). Thus, *q*_*i*_ ∈ *p*_*i*_Ψ(*c*) and *q*_*i*_ ∈ *F*(*c*) since (Ψ, *C*) is a SCPR. Therefore, (*p*_*i*_, *q*_*i*_) ∈ Ψ(*c*), *i* ∈ *I* implies (∨_*i*∈*I*_*p*_*i*_, ∨_*i*∈*I*_*q*_*i*_) ∈ Ψ(*c*). This implies that ∨_*i*∈*I*_*q*_*i*_ ∈ ∨_*i*∈*I*_*p*_*i*_Ψ(*c*). Also, ∨_*i*∈*I*_*q*_*i*_ ∈ *F*(*c*) (as (*F*, *C*) is a soft subquantale). So, ∨_*i*∈*I*_*q*_*i*_ ∈ ∨_*i*∈*I*_*p*_*i*_ ∈ Ψ(*c*)∩*F*(*c*). Hence, $\vee_{i\in I}p_{i}\in {\bar{\Psi }}^{F}\left(c\right)$.

Let $p_{1},\;p_{2}\in {\bar{\Psi }}^{F}\left(c\right)$. Then, *p*_1_Ψ(*c*)∩*F*(*c*) ≠ ∅ and $p_{2}\bar{\Psi }\left(c\right)\cap F\left(c\right)\neq \emptyset$. So, there exists *q*_1_ ∈ *p*_1_Ψ(*c*)∩*F*(*c*) and *q*_2_ ∈ *p*_2_Ψ(*c*)∩*F*(*c*). Thus, *q*_1_ ∈ *p*_1_Ψ(*c*), *q*_1_ ∈ *F*(*c*), *q*_2_ ∈ *p*_2_Ψ(*c*), and *q*_2_ ∈ *F*(*c*) since (Ψ, *C*) is a SCPR. Therefore, (*p*_1_, *q*_1_), (*p*_2_, *q*_2_) ∈ Ψ(*c*) implies (*p*_1_°_1_*q*_1_), (*p*_2_°_2_*q*_2_) ∈ Ψ(*c*). This implies that *q*_1_°_2_*q*_2_ ∈ *p*_1_°*p*_2_Ψ(*c*). Also, *q*_1_°_2_*q*_2_ ∈ (*c*) (as (*F*, *C*) is a soft subquantale). So, *q*_1_°_2_*q*_2_ ∈ *p*_1_°*p*_2_Ψ(*c*)∩*F*(*c*). Hence, $\left(p_{1}{{}^{\circ}}_{1}q_{1}\right)\in {\bar{\Psi }}^{F}\left(c\right)$. This completes the proof.

With the same arguments, the next Theorem 6 can be achieved.

**Theorem 6 .**

Let (Ψ, *C*) be a SCPR over Θ_1_ × Θ_2_. If (*L*, *C*) is a soft subquantale of Θ_1_, then (*L*, *C*) is a *GU*_*p*_*S* subquantale of Θ_2_ w.r.t the foresets.

**Theorem 7 .**

Let (Ψ, *C*) be a soft ∨-complete relation over Θ_1_ × Θ_2_ w.r.t the aftersets. If (*F*, *C*) is a soft left (right) ideal of Θ_2_, then (*F*, *C*) is a *GU*_*p*_*S* left (right) ideal of Θ_1_ w.r.t the aftersets.

**Proof**

Suppose that (*F*, *C*) is a soft left ideal of Θ_2_, then $\emptyset \neq {\bar{\Psi }}^{F}\left(c\right)$ for any *c* ∈ *C*. Let $u_{1},\;u_{2}\in {\bar{\Psi }}^{F}\left(c\right)$. Then, *u*_1_Ψ(*c*)∩*F*(*c*) ≠ ∅ and *u*_2_Ψ(*c*)∩*F*(*c*) ≠ ∅. So, there exists *v*_1_ ∈ *u*_1_Ψ(*c*)∩*F*(*c*) and *v*_2_ ∈ *u*_2_Ψ(*c*)∩*F*(*c*). Thus, *v*_1_ ∈ *u*_1_Ψ(*c*), *v*_1_ ∈ *F*(*c*), *v*_2_ ∈ *u*_2_Ψ(*c*), and *v*_2_ ∈ *F*(*c*) since (Ψ, *C*) is a SCPR. Therefore, (*u*_1_∨*u*_2_, *v*_1_∨*v*_2_) ∈ Ψ(*c*); that is, *v*_1_∨*v*_2_ ∈ (*u*_1_∨*u*_2_)Ψ(*c*). Also, *v*_1_∨*v*_2_ ∈ *F*(*c*) (as (*F*, *C*) is a soft left ideal). So, *v*_1_∨*v*_2_ ∈ (*u*_1_∨*u*_2_)Ψ(*c*)∩*F*(*c*). Hence, $u_{1}\vee u_{2}\in {\bar{\Psi }}^{F}\left(c\right)$.

Now, let *u*_1_,  *u*_2_ ∈ Θ_1_ such that *u*_1_ ≤ *u*_2_ and $u_{2}\in {\bar{\Psi }}^{F}\left(c\right)$. So, $u_{1}\vee u_{2}=u_{2}\in {\bar{\Psi }}^{F}\left(c\right)$. Since $u_{2}\in {\bar{\Psi }}^{F}\left(c\right)$, so there exist *v*_2_ ∈ *u*_2_Ψ(*c*)∩*F*(*c*). Thus, *v*_2_ ∈ *u*_2_Ψ(*c*) and *v*_2_ ∈ *F*(*c*). Since (Ψ, *C*) is a soft ∨-complete relation, therefore, *v*_2_ ∈ *u*_2_Ψ(*c*)*=u*_1_∨*u*_2_Ψ(*c*)*=u*_1_Ψ(*c*)∨*u*_2_Ψ(*c*). This implies that *v*_2_*=s*∨*t*, for some *s* ∈ *u*_1_Ψ(*c*) and *t* ∈ *u*_2_Ψ(*c*). Thus, *s* ≤ *v*_2_ and *v*_2_ ∈ *F*(*c*) imply *s* ∈ *F*(*c*) (as *F*(*c*) is ideal). So, *s* ∈ *u*_1_Ψ(*c*)∩*F*(*c*). Hence, $u_{1}\in {\bar{\Psi }}^{F}\left(c\right)$.

Let *p*,  *x* ∈ Θ_1_ and $x\in {\bar{\Psi }}^{F}\left(c\right)$. Then, *x*Ψ(*c*)∩*F*(*c*) ≠ ∅. So, there exist *q* ∈ *x*Ψ(*c*)∩*F*(*c*). Thus, *q* ∈ *x*Ψ(*c*) and *q* ∈ *F*(*c*). Since (*F*, *C*) is a soft left ideal so, *y*∘_2_*q* ∈ *F*(*c*) for any *y* ∈ *p*Ψ(*c*)⊆Θ_2_. This implies that (*p*, *y*) ∈ Ψ(*c*). So, (*p*∘_1_*x*, *y*∘_2_*q*) ∈ Ψ(*c*); that is, *y*∘_2_*q* ∈ *p*∘_1_*x*Ψ(*c*). So, *y*∘_2_*q* ∈ *p*∘_1_*x*Ψ(*c*)∩*F*(*c*). Hence, $p\circ_{1}x\in {\bar{\Psi }}^{F}\left(c\right)$. Similarly, we can show that $x\circ_{1}p\in {\bar{\Psi }}^{F}\left(c\right)$.

**Theorem 8 .**

Let (Ψ, *C*) be a SCTR over Θ_1_ × Θ_2_ w.r.t the aftersets. If (*F*, *C*) is a soft prime ideal of Θ_2_, then (*F*, *C*) is a *GU*_*p*_*S* prime ideal of Θ_1_ w.r.t the aftersets.

**Proof**

Assume that (*F*, *C*) is a soft prime ideal of Θ_2_, then $\emptyset \neq {\bar{\Psi }}^{F}\left(c\right)$ for any *c* ∈ *C*. Then, by Theorem 5, (*F*, *C*) is generalized upper soft ideal of Θ_1_. Let *p*_1_,  *p*_2_ ∈ Θ_1_ such that $p_{1}\circ_{1}p_{2}\in {\bar{\Psi }}^{F}\left(c\right)$. Then, (*p*_1_∘_1_*p*_2_)Ψ(*c*)∩*F*(*c*) ≠ ∅. So, there exist *q* ∈ (*p*_1_∘_1_*p*_2_)Ψ(*c*)∩*F*(*c*). This implies that *q* ∈ (*p*_1_∘_1_*p*_2_)Ψ(*c*) and *q* ∈ *F*(*c*). Since (Ψ, *C*) is a SCTR, *q* ∈ (*p*_1_∘_1_*p*_2_)Ψ(*c*)*=p*_1_Ψ(*c*)∘_2_*p*_2_Ψ(*c*). Thus, *q=c*∘_2_*d* for some *c* ∈ *p*_1_Ψ(*c*) and *d* ∈ *p*_2_Ψ(*c*). Thus, *c*∘_2_*d* ∈ *F*(*c*) and (*F*, *C*) is a soft prime ideal of Θ_2_ so, *c* ∈ *F*(*c*) or *d* ∈ *F*(*c*). Thus, *c* ∈ *p*_1_Ψ(*c*)∩*F*(*c*) or *d* ∈ *p*_2_Ψ(*c*)∩*F*(*c*). Hence, $p_{1}\in {\bar{\Psi }}^{F}\left(c\right)$ or $p_{2}\in {\bar{\Psi }}^{F}\left(c\right)$.

**Theorem 9 .**

Let (Ψ, *C*) be a SCTR over Θ_1_ × Θ_2_ w.r.t the aftersets. If (*F*, *C*) is a soft semiprime ideal of Θ_2_, then (*F*, *C*) is a *GU*_*p*_*S* semiprime ideal of Θ_1_ w.r.t the aftersets.

**Proof**

Assume that (*F*, *C*) is a soft semiprime ideal of Θ_2_, then $\emptyset \neq {\bar{\Psi }}^{F}\left(c\right)$ for any *c* ∈ *C*. Then, by Theorem 5, (*F*, *C*) is generalized upper soft ideal of Θ_1_. Let *p*_1_ ∈ Θ_1_ such that $p_{1}\circ_{1}p_{1}\in {\bar{\Psi }}^{F}\left(c\right)$. Then, (*p*_1_∘_1_*p*_1_)Ψ(*c*)∩*F*(*c*) ≠ ∅. So, there exist *q* ∈ (*p*_1_∘_1_*p*_1_)Ψ(*c*)∩*F*(*c*). This implies that *q* ∈ (*p*_1_∘_1_*p*_1_)Ψ(*c*) and *q* ∈ *F*(*c*). Since (Ψ, *C*) is a SCTR, *q* ∈ (*p*_1_∘_1_*p*_1_)Ψ(*c*)*=p*_1_Ψ(*c*)∘_2_ *p*_1_Ψ(*c*). Thus, *q=c* ∘_2_ *c* for some *c* ∈ *p*_1_Ψ(*c*). Thus, *c*∘_2_*c* ∈ *F*(*c*) and (*F*, *C*) is a soft semiprime ideal of Θ_2_ so, *c* ∈ *F*(*c*). Thus, *c* ∈ *p*_1_Ψ(*c*)∩*F*(*c*). Hence, $p_{1}\in {\bar{\Psi }}^{F}\left(c\right)$.

**Theorem 10 .**

Let (Ψ, *C*) be a SCTR over Θ_1_ × Θ_2_ w.r.t the aftersets. If (*F*, *C*) is a soft primary ideal of Θ_2_, then (*F*, *C*) is a *GU*_*p*_*S* primary ideal of Θ_1_ w.r.t the aftersets.

**Proof**

Assume that (*F*, *C*) is a soft primary ideal of Θ_2_, then $\emptyset \neq {\bar{\Psi }}^{F}\left(c\right)$ for any c ∈ *C*. Then, by Theorem 5, (*F*, *C*) is generalized upper soft ideal of Θ_1_. Let *p*_1_,  *p*_2_ ∈ Θ_1_ such that *p*_1_∘_1_*p*_2_∈${\bar{\Psi }}^{F}\left(c\right)$ and $p_{1}\notin {\bar{\Psi }}^{F}\left(c\right)$. Then, (*p*_1_∘_1_*p*_2_)Ψ(*c*)∩*F*(*c*) ≠ ∅. So, there exist *q* ∈ (*p*_1_∘_1_*p*_2_)Ψ(*c*)∩*F*(*c*). This implies that *q* ∈ (*p*_1_∘_1_*p*_2_)Ψ(*c*) and *q* ∈ *F*(*c*). Since (Ψ, *C*) is a SCTR, *q* ∈ (*p*_1_∘_1_*p*_2_)Ψ(*c*)*=p*_1_Ψ(*c*)∘_2_*p*_2_Ψ(*c*). Thus, *q=c*∘_2_*d* for some *c* ∈ *p*_1_Ψ(*c*) and *d* ∈ *p*_2_Ψ(*c*). Thus, *c*∘_2_*d* ∈ *F*(*c*) and (*F*, *C*) is a soft primary ideal of Θ_2_ so *d*^*n*^ ∈ *F*(*c*) for some *n* ∈ *ℕ*. Also, *d*^*n*^ ∈ *p*_2_^*n*^Ψ(*c*) for *n* ∈ *ℕ*. Thus, *d*^*n*^ ∈ *p*_2_^*n*^Ψ(*c*)∩*F*(*c*). Hence, $p_{2}^{n}\in {\bar{\Psi }}^{F}\left(c\right)$.

**Remark 2 .**

In general, the converse of the above theorem is not true. We will present examples to justify our claim as follows.

**Example 2 .**

**Figure 2:**
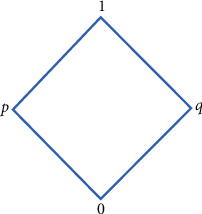
Illustration of Θ_1_.

**Figure 3:**
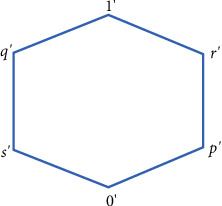
Illustration of Θ_2_.

Let Θ_1_*=*{0, *p*, *q*, 1} and Θ_2_*=*{0′, *s*′, *q*′, *p*′, 1′, *r*′} be two complete lattices described in Figures 2 and 3, respectively.

We define ∘_1_ and ∘_2_ the associative binary operation on Θ_1_ and Θ_2_, respectively, as shown in Tables 2 and 3. Then, and are quantales.

(1) Let *C=*{*c*_1_, *c*_2_} and define SBR (Ψ, *C*) over Θ_1_ × Θ_2_ by the rule
  (8) $$\begin{array}{c} \Psi \left(c_{1}\right)=\left\{\begin{array}{c} \left(0,q^{\prime }\right),\left(0,0^{\prime }\right),\left(p,s^{\prime }\right),\left(0,s^{\prime }\right),\left(q,p^{\prime }\right),\\ \left(p,r^{\prime }\right),\left(0,1^{\prime }\right),\left(0,p^{\prime }\right),\left(p,1^{\prime }\right),\left(q,s^{\prime }\right),\\ \left(q,1^{\prime }\right),\left(p,q^{\prime }\right),\left(q,r^{\prime }\right),\left(1,r^{\prime }\right),\left(1,s^{\prime }\right),\\ \left(1,1^{\prime }\right),\left(0,r^{\prime }\right) \end{array}\right\},\\ \Psi \left(c_{2}\right)=\left\{\begin{array}{c} \left(0,r^{\prime }\right),\left(0,0^{\prime }\right),\left(0,p^{\prime }\right),\left(0,1^{\prime }\right),\left(0,s^{\prime }\right),\\ \left(p,r^{\prime }\right),\left(0,q^{\prime }\right),\left(p,s^{\prime }\right),\left(p,1^{\prime }\right),\left(p,q^{\prime }\right) \end{array}\right\}. \end{array}$$
Then, (Ψ, *C*) is SCPR. The aftersets with respect to Ψ(*c*_1_) and Ψ(*c*_2_) are given as follows:
  (9) $$\begin{array}{c} 0\Psi \left(c_{1}\right)=\left\{0^{\prime },s^{\prime },p^{\prime },q^{\prime },r^{\prime },1^{\prime }\right\},\\ 0\Psi \left(c_{2}\right)=\left\{0^{\prime },s^{\prime },p^{\prime },r^{\prime },1^{\prime },q^{\prime }\right\},\\ p\Psi \left(c_{1}\right)=\left\{r^{\prime },s^{\prime },1^{\prime },q^{\prime }\right\},\\ p\Psi \left(c_{2}\right)=\left\{r^{\prime },s^{\prime },1^{\prime },q^{\prime }\right\},\\ q\Psi \left(c_{2}\right)=\emptyset ,\\ 1\Psi \left(c_{1}\right)=\left\{r^{\prime },s^{\prime },1^{\prime }\right\}\\ 1\Psi \left(c_{2}\right)=\emptyset . \end{array}$$
Define soft set (*F*, *C*) over Θ_2_ by the rule
  (10) $$\begin{array}{c} F\left(c_{1}\right)=\left\{r^{\prime },s^{\prime }\right\},\\ F\left(c_{2}\right)=\left\{q^{\prime },r^{\prime }\right\}. \end{array}$$
Then, (*F*, *C*) is not a soft subquantale of Θ_2_. But ${\bar{\Psi }}^{F}\left(c_{1}\right)=\left\{0,p,q,1\right\}$ and ${\bar{\Psi }}^{F}\left(c_{2}\right)=\left\{0,p\right\}$ are subquantale of Θ_1_. So (*F*, *C*) is a *GU*_*p*_*SS*_Θ_ of Θ_1_ w.r.t the aftersets.
Foresets with respect to Ψ(*c*_1_) and Ψ(*c*_2_) are given as follows:
  (11) $$\begin{array}{c} \Psi \left(c_{1}\right)0^{\prime }=\left\{0\right\},\\ \Psi \left(c_{2}\right)0^{\prime }=\left\{0\right\},\\ \Psi \left(c_{1}\right)p^{\prime }=\left\{0,q\right\},\\ \Psi \left(c_{2}\right)p^{\prime }=\left\{0\right\},\\ \Psi \left(c_{1}\right)q^{\prime }=\left\{0,p\right\},\\ \Psi \left(c_{2}\right)q^{\prime }=\left\{0,p\right\},\\ \Psi \left(c_{1}\right)r^{\prime }=\left\{0,p,q,1\right\},\\ \Psi \left(c_{2}\right)r^{\prime }=\left\{0,p\right\},\\ \Psi \left(c_{1}\right)s^{\prime }=\left\{0,p,q,1\right\},\\ \Psi \left(c_{2}\right)s^{\prime }=\left\{0,p\right\},\\ \Psi \left(c_{1}\right)1^{\prime }=\left\{0,p,q,1\right\},\\ \Psi \left(c_{2}\right)1^{\prime }=\left\{0,p\right\}. \end{array}$$
Define soft set (*L*, *C*) over Θ_1_ by the rule
  (12) $$\begin{array}{c} L\left(c_{1}\right)=\left\{p,\;q\right\},\\ L\left(c_{2}\right)=\left\{0,p,q\right\}. \end{array}$$
Then, (*L*, *C*) is not a soft subquantale of Θ_1_. But Ψ¯Lc1=p′,q′,r′,s′,1′ and Ψ¯Lc2=0′,p′,q′,r′,s′,1′ are subquantale of Θ_2_. So, (*L*, *C*) is a *GU*_*p*_*S* subquantale of Θ_2_ w.r.t the foresets.
(2) Now, let *C=*{*c*_1_, *c*_2_} and define SBR (Ψ, *C*) over Θ_1_ × Θ_2_ by the rule

(13) $$\begin{array}{c} \Psi \left(c_{1}\right)=\left\{\left(q,0^{\prime }\right),\left(p,0^{\prime }\right),\left(q,p^{\prime }\right),\left(0,p^{\prime }\right),\left(1,0^{\prime }\right),\left(1,p^{\prime }\right),\left(p,p^{\prime }\right),\left(0,0^{\prime }\right)\right\}. \end{array}$$

(14) $$\begin{array}{c} \Psi \left(c_{2}\right)=\left\{\left(p,q^{\prime }\right),\left(0,s^{\prime }\right),\left(0,q^{\prime }\right),\left(q,s^{\prime }\right),\left(1,\;s^{\prime }\right),\left(1,q^{\prime }\right),\left(q,q^{\prime }\right),\left(p,s^{\prime }\right)\right\}. \end{array}$$

Aftersets with respect to Ψ(*c*_1_) and Ψ(*c*_2_) are given as follows:

(15) $$\begin{array}{c} 0\Psi \left(c_{1}\right)=\left\{0^{\prime },p^{\prime }\right\},\\ 0\Psi \left(c_{2}\right)=\left\{s^{\prime },q^{\prime }\right\},\\ p\Psi \left(c_{1}\right)=\left\{0^{\prime },p^{\prime }\right\},\\ p\Psi \left(c_{2}\right)=\left\{s^{\prime },q^{\prime }\right\},\\ q\Psi \left(c_{1}\right)=\left\{0^{\prime },p^{\prime }\right\},\\ q\Psi \left(c_{2}\right)=\left\{s^{\prime },q^{\prime }\right\},\\ 1\Psi \left(c_{1}\right)=\left\{0^{\prime },p^{\prime }\right\},\\ 1\Psi \left(c_{2}\right)=\left\{s^{\prime },q^{\prime }\right\}. \end{array}$$

Then, (Ψ, *C*) is ∨-complete relation over Θ_1_ × Θ_2_ w.r.t the aftersets. Define soft set (*F*, *C*) over Θ_2_ by the rule

(16) $$\begin{array}{c} F\left(c_{1}\right)=\left\{s^{\prime },p^{\prime }\right\},\\ F\left(c_{2}\right)=\left\{s^{\prime },r^{\prime }\right\}. \end{array}$$

Then, (*F*, *C*) is not a soft ideal of Θ_2_. But ${\bar{\Psi }}^{F}\left(c_{1}\right)=\left\{0,p,q,1\right\}$ and ${\bar{\Psi }}^{F}\left(c_{2}\right)=\left\{0,p,q,1\right\}$ are ideal of Θ_1_. So, (*F*, *C*) is a *GU*_*p*_*S* ideal of Θ_1_ w.r.t the aftersets.

Now, define SBR (Ψ, *C*) over Θ_1_ × Θ_2_ by the rule

(17) $$\begin{array}{c} \Psi \left(c_{1}\right)=\left\{\begin{array}{c} \left(0,q^{\prime }\right),\left(p,q^{\prime }\right),\left(0,0^{\prime }\right),\left(p,0^{\prime }\right),\\ \left(p,s^{\prime }\right),\left(p,r^{\prime }\right),\left(0,1^{\prime }\right),\left(0,r^{\prime }\right),\\ \left(p,p^{\prime }\right),\left(0,s^{\prime }\right),\left(p,1^{\prime }\right),\left(0,p^{\prime }\right) \end{array}\right\} \end{array}$$

(18) $$\begin{array}{c} \Psi \left(c_{2}\right)=\left\{\begin{array}{c} \left(0,0^{\prime }\right),\left(q,r^{\prime }\right),\left(q,q^{\prime }\right),\left(0,s^{\prime }\right),\\ \left(0,1^{\prime }\right),\left(q,0^{\prime }\right),\left(0,r^{\prime }\right),\left(q,p^{\prime }\right),\\ \left(0,p^{\prime }\right),\left(q,s^{\prime }\right),\left(q,1^{\prime }\right),\left(0,q^{\prime }\right) \end{array}\right\} \end{array}$$

Foresets with respect to Ψ(*c*_1_) and Ψ(*c*_2_) are given as follows:

(19) $$\begin{array}{c} \Psi \left(c_{1}\right)0^{\prime }=\left\{p,0\right\},\\ \Psi \left(c_{2}\right)0^{\prime }=\left\{q,0\right\},\\ \Psi \left(c_{1}\right)p^{\prime }=\left\{p,0\right\},\\ \Psi \left(c_{2}\right)p^{\prime }=\left\{0,q\right\},\\ \Psi \left(c_{1}\right)q^{\prime }=\left\{p,0\right\},\\ \Psi \left(c_{2}\right)q^{\prime }=\left\{q,0\right\},\\ \Psi \left(c_{1}\right)r^{\prime }=\left\{0,p\right\},\\ \Psi \left(c_{2}\right)r^{\prime }=\left\{q,0\right\},\\ \Psi \left(c_{1}\right)s^{\prime }=\left\{0,p\right\},\\ \Psi \left(c_{2}\right)s^{\prime }=\left\{q,0\right\},\\ \Psi \left(c_{1}\right)1^{\prime }=\left\{0,p\right\},\\ \Psi \left(c_{2}\right)1^{\prime }=\left\{0,q\right\}. \end{array}$$

Then, (Ψ, *C*) is soft ∨-complete relation over Θ_1_ × Θ_2_ w.r.t the foresets. Define soft set (*L*, *C*) over Θ_1_ by the rule

(20) $$\begin{array}{c} L\left(c_{1}\right)=\left\{p,q\right\},\\ L\left(c_{2}\right)=\left\{0,p,q\right\}. \end{array}$$

Then, (*L*, *C*) is not a soft ideal of Θ_1_. But Ψ¯Lc1=0′,p′,q′,r′,s′,1′ and Ψ¯Lc2=0′,p′,q′,r′,s′,1′ are ideal of Θ_2_. So, (*L*, *C*) is a *GU*_*p*_*S* ideal of Θ_2_ w.r.t the foresets.

Similar examples can be presented to justify that converse of Theorems 11to 13 is not true.

**Definition 13 .**

Let (Ψ, *C*) be a SBR over Θ_1_ × Θ_2_. Consider the soft set (*M*, *C*) over Θ_2_, if *L*_appr_$\left({\underline{\Psi }}^{M},C\right)$ is a soft subquantale of Θ_1_, then (*M*, *C*) is called generalized lower soft (*GL*_*W*_*S*) subquantale of Θ_1_ w.r.t the aftersets. If *L*_appr_$\left({\underline{\Psi }}^{M},C\right)$ is a soft ideal (prime ideal, semiprime ideal, and primary ideal) of Θ_1_, then (*M*, *C*) is called *GU*_*p*_*S* ideal (prime ideal, semiprime ideal, and primary ideal) of Θ_1_ w.r.t the aftersets.

**Definition 14 .**

Let (Ψ, *C*) be a SBR over Θ_1_ × Θ_2_. Consider the soft set (*L*, *C*) over Θ_1_, if *L*_appr_Ψ¯L,C is a soft subquantale of Θ_2_, then (*L*, *C*) is called *GL*_*W*_*S* subquantale of Θ_2_ w.r.t the foresets. If *L*_appr_Ψ¯L,C is a soft ideal (prime ideal, semiprime ideal, and primary ideal) of Θ_2_, then (*L*, *C*) is called *GU*_*p*_*S* ideal (prime ideal, semiprime ideal, and primary ideal) of Θ_2_ w.r.t the foresets.

**Theorem 11 .**

Let (Ψ, *C*) be a SCTR over Θ_1_ × Θ_2_ w.r.t the aftersets. If (*M*, *C*) is a soft subquantale of Θ_2_, then (*M*, *C*) is a *GL*_*W*_*S* subquantale of Θ_1_ w.r.t the aftersets.

**Proof**

Suppose that (*M*, *C*) is a soft subquantale of Θ_2_ and ${\underline{\Psi }}^{M}\left(c\right)\neq \emptyset$ for any c ∈ *C*. Let $u_{i}\in {\underline{\Psi }}^{M}\left(c\right),\;i\in I$. Then, *u*_*i*_Ψ(*c*)⊆*M*(*c*). Since (Ψ, *C*) is a SCTR, therefore, ∨_*i*∈*I*_(*u*_*i*_Ψ(*c*))*=*(∨_*i*∈*I*_*u*_*i*_)Ψ(*c*)⊆*M*(*c*). Hence, $\vee_{i\in I}u_{i}\in {\underline{\Psi }}^{M}\left(c\right)$.

Now, let $u_{1},\;u_{2}\in {\underline{\Psi }}^{M}\left(c\right)$. Then, *u*_1_Ψ(*c*)⊆*M*(*c*) and *u*_2_Ψ(*c*)⊆*M*(*c*). Since (Ψ, *C*) is a SCTR and (*M*, *C*) is a soft subquantale, therefore, *u*_1_Ψ(*c*)∘_2_*u*_2_Ψ(*c*)⊆*M*(*c*)∘_2_*M*(*c*) implies (*u*_1_∘_1_*u*_2_)Ψ(*c*)⊆*M*(*c*). Hence, $u_{1}\circ_{1}u_{2}\in {\underline{\Psi }}^{M}\left(c\right)$.

With the same arguments, next Theorem 12 can be achieved.

**Theorem 12 .**

Let (Ψ, *C*) be a SCTR over Θ_1_ × Θ_2_ w.r.t the foresets. If (*L*, *C*) is a soft subquantale of Θ_1_, then (*L*, *C*) is a *GL*_*W*_*S* subquantale of Θ_2_ w.r.t the foresets.

**Theorem 13 .**

Let (Ψ, *C*) be a SCTR over Θ_1_ × Θ_2_ w.r.t the aftersets. If (*M*, *C*) is a soft ideal of Θ_2_, then (*M*, *C*) is a *GL*_*W*_*S* ideal of Θ_1_ w.r.t the aftersets.

**Proof**

Suppose that (*M*, *C*) is a soft ideal of Θ_2_ and ${\underline{\Psi }}^{M}\left(c\right)\neq \emptyset$ for any c ∈ *C*. Let $u_{1},\;u_{2}\in {\underline{\Psi }}^{M}\left(c\right)$. Then, *u*_1_Ψ(*c*)⊆*M*(*c*) and *u*_2_Ψ(*c*)⊆*M*(*c*). Since (Ψ, *C*) is a SCTR and (*M*, *C*) is a soft ideal of Θ_2_ so *u*_1_Ψ(*c*)∨*u*_2_Ψ(*c*)*=*(*u*_1_∨*u*_2_)Ψ(*c*)⊆*M*(*c*)∨*M*(*c*); that is, (*u*_1_∨*u*_2_)Ψ(*c*)⊆*M*(*c*) Hence, $u_{1}\vee u_{2}\in {\underline{\Psi }}^{M}\left(c\right)$.

Now, let *u*_1_,  *u*_2_ ∈ Θ_1_ such that *u*_1_ ≤ *u*_2_ and $u_{2}\in {\underline{\Psi }}^{M}\left(c\right)$. So, $u_{1}\vee u_{2}=u_{2}\in {\underline{\Psi }}^{M}\left(c\right)$. Let, *v*_1_ ∈ *u*_1_Ψ(*c*) and *v*_2_ ∈ *u*_2_Ψ(*c*)⊆*M*(*c*). So, *v*_1_∨*v*_2_ ∈ (*u*_1_∨*u*_2_)Ψ(*c*), that is, *v*_1_∨*v*_2_ ∈ *u*_2_Ψ(*c*)⊆*M*(*c*). Since *M*(*c*) is ideal so *v*_1_ ≤ *v*_1_∨*v*_2_ ∈ *M*(*c*) implies *v*_1_ ∈ *M*(*c*). Thus, *u*_1_Ψ(*c*)⊆*M*(*c*). Hence, $u_{1}\in {\underline{\Psi }}^{M}\left(c\right)$.

Now, let *u*,  *y* ∈ Θ_1_ and $y\in {\underline{\Psi }}^{M}\left(c\right)$. Then, ∅≠*y*Ψ(*c*)⊆*M*(*c*). Consider *v*_1_∈(*u*∘_1_*y*)Ψ(*c*) since (Ψ, *C*) is a SCTR so *v*_1_ ∈ *u* Ψ(*c*)∘_2_*y*Ψ(*c*). Thus, *v*_1_*=c*∘_2_*d* for some *c* ∈ *u*Ψ(*c*) and *d* ∈ *y*Ψ(*c*). But *y*Ψ(*c*)⊆*M*(*c*) so *d* ∈ *M*(*c*) and (*M*, *C*) is a soft ideal of Θ_2_; therefore, *c*∘_2_*d* ∈ *M*(*c*), that is, *v*_1_ ∈ *M*(*c*). Thus, (*u*∘_1_*y*)Ψ(*c*)⊆*M*(*c*). Hence, $u\circ_{1}y\in {\underline{\Psi }}^{M}\left(c\right)$. Similarly, we can show that $y\circ_{1}u\in {\underline{\Psi }}^{M}\left(c\right)$.

**Theorem 14 .**

Let (Ψ, *C*) be a SCTR over Θ_1_ × Θ_2_ w.r.t the aftersets. If (*M*, *C*) is a soft prime ideal of Θ_2_, then (*M*, *C*) is a *GL*_*W*_*S* prime ideal of Θ_1_ w.r.t the aftersets.

**Proof**

Assume that (*M*, *C*) is a soft prime ideal of Θ_2_ and ${\underline{\Psi }}^{M}\left(c\right)\neq \emptyset$ for any c ∈ *C*. Then, by Theorem 4.19, (*M*, *C*) is *GL*_*W*_*S* ideal of Θ_1_. Let *u*_1_,  *u*_2_ ∈ Θ_1_ such that $u_{1}\circ_{1}u_{2}\in {\underline{\Psi }}^{M}\left(c\right)$. Then, (*u*_1_∘_1_*u*_2_)Ψ(*c*)⊆*M*(*c*). Consider *v* ∈ (*u*_1_∘_1_*u*_2_)Ψ(*c*)⊆*M*(*c*). Since (Ψ, *C*) is a SCTR, *v* ∈ (*u*_1_∘_1_*u*_2_)Ψ(*c*)*=u*_1_Ψ(*c*)∘_2_*u*_2_Ψ(*c*). Thus, *v=c*∘_2_*d* for some *c* ∈ *u*_1_Ψ(*c*) and *d* ∈ *u*_2_Ψ(*c*). This implies that *v=c*∘_2_*d* ∈ *M*(*c*). As (*M*, *C*) is a soft prime ideal so, *c* ∈ *M*(*c*) or *d* ∈ *M*(*c*). Thus, *c* ∈ *u*_1_Ψ(*c*)⊆*M*(*c*) or *d* ∈ *u*_2_Ψ(*c*)⊆*M*(*c*). Hence, $u_{1}\in {\underline{\Psi }}^{M}\left(c\right)$ or $u_{2}\in {\underline{\Psi }}^{M}\left(c\right)$.

**Theorem 15 .**

Let (Ψ, *C*) be a SCTR over Θ_1_ × Θ_2_ w.r.t the aftersets. If (*M*, *C*) is a soft semiprime ideal of Θ_2_, then (*M*, *C*) is a *GL*_*W*_*S* semiprime ideal of Θ_1_ w.r.t the aftersets.

**Proof**

Assume that (*M*, *C*) is a soft semiprime ideal of Θ_2_ and ${\underline{\Psi }}^{M}\left(c\right)\neq \emptyset$ for any c ∈ *C*. Then, by Theorem 14,(*M*, *C*) is *GL*_*W*_*S* ideal of Θ_1_. Let *u* ∈ Θ_1_ such that $u\circ_{1}u\in {\underline{\Psi }}^{M}\left(c\right)$. Then, (*u*∘_1_*u*)Ψ(*c*)⊆*M*(*c*). Let *v* ∈ *u*Ψ(*c*). As (Ψ, *C*) is a SCTR so *v*∘_2_*v* ∈ (*u*∘_1_*u*)Ψ(*c*)⊆*M*(*c*). Since (*M*, *C*) is a soft semiprime ideal, *v*∘_2_*v* ∈ *M*(*c*) implies *v* ∈ *M*(*c*). Thus, *u*Ψ(*c*)⊆*M*(*c*). Hence, $u\in {\underline{\Psi }}^{M}\left(c\right)$.

**Theorem 16 .**

Let (Ψ, *C*) be a SCTR over Θ_1_ × Θ_2_ w.r.t the aftersets. If (*M*, *C*) is a soft primary ideal of Θ_2_, then (*M*, *C*) is a *GL*_*W*_*S* primary ideal of Θ_1_ w.r.t the aftersets.

**Proof**

Suppose that (*M*, *C*) is a soft primary ideal of Θ_2_ and $\emptyset \neq {\underline{\Psi }}^{M}\left(c\right)$ for any c ∈ *C*. Then, by Theorem 4.19., (*M*, *C*) is a *GL*_*W*_*S* ideal of Θ_1_. Let *u*_1_,  *u*_2_ ∈ Θ_1_ such that $u_{1}\circ_{1}u_{2}\in {\underline{\Psi }}^{M}\left(c\right)$ and $u_{1}\notin {\underline{\Psi }}^{M}\left(c\right)$. Then, (*u*_1_∘_1_*u*_2_)Ψ(*c*)⊆*M*(*c*). Let *v* ∈ (*u*_1_∘_1_*u*_2_)Ψ(*c*). Since (Ψ, *C*) is a SCTR, *v* ∈ *u*_1_Ψ(*c*)∘_2_*u*_2_Ψ(*c*). Thus, *v=c*∘_2_*d* for some *c* ∈ *u*_1_Ψ(*c*) and *d* ∈ *u*_2_Ψ(*c*). Thus, *d*^*n*^ ∈ *u*_2_^*n*^Ψ(*c*) for some *n* ∈ *ℕ*. Also, *c*∘_2_*d* ∈ *M*(*c*). As (*M*, *C*) is a soft primary ideal, *c* ∉ *M*(*c*) and *d*^*n*^ ∈ *M*(*c*). Thus, *u*_2_^*n*^Ψ(*c*)⊆*M*(*c*). Hence, $u_{2}^{n}\in {\underline{\Psi }}^{M}\left(c\right)$ for some *n* ∈ *ℕ*.

**Remark 3 .**

One can find examples like Example 2 to show that converse of Theorems 11to 16 is not true.

## 5. Relationship between Soft Quantale Homomorphism and Their Approximation

In this section, we define soft weak quantale homomorphism (SWQH), and then, we established the relationship between homomorphic images and their approximation by SBR.

**Definition 15 (see [4]).**

A function *η* : Θ_1_⟶Θ_2_ is called weak quantale homomorphism (WQH) if *η*(*p*∘_1_*q*)*=η*(*p*)∘_2_*η*(*q*) and *η*(*p*∨*q*)*=η*(*p*)∨*η*(*q*), where (Θ_1_, ∘_1_) and (Θ_2_, ∘_2_) are quantales. If *η* is one-one, then *η* is monomorphism. If *η* is onto, then *η* is called epimorphism, and if *η* is bijective, then *η* is called isomorphism between (Θ_1_, ∘_1_) and (Θ_2_, ∘_2_).

**Definition 16 .**

Let (*H*, *C*_1_) be a soft quantale over Θ_1_ and (*F*, *C*_2_) be a soft quantale over Θ_2_. Then, (*H*, *C*_1_) is said to soft weak homomorphic to (*F*, *C*_2_) if there exist ordered pair of functions (*η*, *ζ*) satisfies the following

1. *η* : Θ_1_⟶Θ_2_ is onto WQH, that is, *η*(*p*∘_1_*q*)*=η*(*p*)∘_2_*η*(*q*) and *η*(*p*∨*q*)*=η*(*p*)∨*η*(*q*)
2. *ζ* : *C*_1_⟶*C*_2_ is surjective
3. *η*(*H*(*c*_1_))*=F*(*ζ*(*c*_1_)), ∀*c*_1_ ∈ *C*_1_

The ordered pair (*η*, *ζ*) of functions is SWQH. If in ordered pair (*η*, *ζ*) both *η* and *ζ* are one-to-one functions, then (*H*, *C*_1_) is said to soft weak isomorphic to (*F*, *C*_2_) and (*η*, *ζ*) is called SWQI.

**Lemma 1 .**

Let (*H*, *C*_1_) be soft weak homomorphic to (*F*, *C*_2_) with SWQH (*η*, *ζ*). Let (Ψ_2_, *C*_3_) be a SBR over Θ_2_ and (*H*_1_, *C*_1_′)⊆(*H*, *C*_1_). Define Ψ_1_(*c*_3_)*=*{}(*x*,  *y*)∈Θ_1_ × Θ_1_:(*η*(*x*), *η*(*y*)) ∈ Ψ_2_(*c*_3_){} be a SBR over Θ_1_. Then, the following holds:

1. (Ψ_1_, *C*_3_) is SCPR if (Ψ_2_, *C*_3_) is SCPR
2. If (*η*, *ζ*) is SWQI and (Ψ_2_, *C*_3_) is SCPR w.r.t the aftersets (w.r.t the foresets), then (Ψ_1_, *C*_3_) is SCPR w.r.t the aftersets (w.r.t the foresets)
3. ηΨ¯1H1c3=Ψ¯2ηH1c3
4. ηΨ¯1H1c3⊆${\underline{\Psi }}_{2}^{\eta \left(H_{1}\right)}\left(c_{3}\right)$ and if (*η*, *ζ*) is SWQI, then ηΨ¯1H1c3=${\underline{\Psi }}_{2}^{\eta \left(H_{1}\right)}\left(c_{3}\right)$
5. Let (*η*, *ζ*) be a SWQI. Then, ηx∈ηΨ¯1H1c3⇔x∈Ψ¯1H1c3 and ηx∈ηΨ¯1H1c3⇔x∈Ψ¯1H1c3

**Proof**

1. and (2) are obvious
2. Suppose (*H*_1_, *C*_1_′)⊆(*H*, *C*_1_) and for any *c*_3_ ∈ *C*_3_, z∈ηΨ¯1H1c3 for some *z* ∈ Θ_2_. Then, there exist *a* ∈ Θ_1_ such that a∈Ψ¯1H1c3 and *η*(*a*)*=z*. Thus, *x* ∈ *a*Ψ_1_(*c*_3_)∩*H*_1_(*c*_1_′). So, (*a*, *x*) ∈ Ψ_1_(*c*_3_) and *x* ∈ *H*_1_(*c*_1_′). Thus, (*η*(*a*), *η*(*x*)) ∈ Ψ_2_(*c*_3_), that is, *η*(*x*) ∈ *η*(*a*)Ψ_2_(*c*_3_). Also, *η*(*x*) ∈ *η*(*H*_1_(*c*_1_′)). So, *η*(*a*)Ψ_2_(*c*_3_)∩*η*(*H*_1_(*c*_1_′))≠∅. This implies that $\eta \left(a\right)\in {\bar{\Psi }}_{2}^{\eta \left(H_{1}\right)}\left(c_{3}\right)$. Hence, ηΨ¯1H1c3⊆${\bar{\Psi }}_{2}^{\eta \left(H_{1}\right)}\left(c_{3}\right)$.
3. Now, let $w\in {\bar{\Psi }}_{2}^{\eta \left(H_{1}\right)}\left(c_{3}\right)$. Then, *w*Ψ_2_(*c*_3_)∩*η*(*H*_1_(*c*_1_′)) ≠ ∅. This implies that *y* ∈ *w*Ψ_2_(*c*_3_)∩*η*(*H*_1_(*c*_1_′)). Thus, *y* ∈ *w*Ψ_2_(*c*_3_) and *y* ∈ *η*(*H*_1_(*c*_1_′)). This implies that there exists *x* ∈ *H*_1_(*c*_1_′)⊆Θ_1_ and *x*_1_ ∈ Θ_1_ such that *η*(*x*)*=y* and *η*(*x*_1_)*=w*. So, (*w*, *y*)*=*(*η*(*x*_1_), *η*(*x*))∈Ψ_2_(*c*_3_). This implies that (*x*_1_, *x*) ∈ Ψ_1_(*c*_3_). So, *x* ∈ *x*_1_Ψ_1_(*c*_3_)∩*H*_1_(*c*_1_′). Thus, x1∈Ψ¯1H1c3. So, w=ηx1∈ηΨ¯1H1c3. Hence, ${\bar{\Psi }}_{2}^{\eta \left(H_{1}\right)}\left(c_{3}\right)\subseteq$ηΨ¯1H1c3. Consequently, ηΨ¯1H1c3=${\bar{\Psi }}_{2}^{\eta \left(H_{1}\right)}\left(c_{3}\right)$.
4. Suppose (*H*_1_, *C*_1_′)⊆(*H*, *C*_1_) and for any *c*_3_ ∈ *C*_3_, z∈ηΨ¯1H1c3 for some *z* ∈ Θ_2_. Then, there exist *a* ∈ Θ_1_ such that a∈Ψ¯1H1c3 and *η*(*a*)*=z*. Thus, *a*Ψ_1_(*c*_3_)⊆*H*_1_(*c*_1_′). Let *x* ∈ *z*Ψ_2_(*c*_3_). Then, there exist *y* ∈ Θ_1_ such that *η*(*y*)*=x*. So, *η*(*y*) ∈ *η*(*a*)Ψ_2_(*c*_3_), that is, (*η*(*a*),  *η*(*y*)) ∈ Ψ_2_(*c*_3_). So, (*a*,  *y*) ∈ Ψ_1_(*c*_3_), that is, *y* ∈ *a*Ψ_1_(*c*_3_)⊆*H*_1_(*c*_1_′). Thus, *η*(*y*)∈*η*(*H*_1_(*c*_1_′)). So, *η*(*a*)Ψ_2_(*c*_3_)⊆*η*(*H*_1_(*c*_1_′)). Thus, $z=\eta \left(a\right)\in {\underline{\Psi }}_{2}^{\eta \left(H_{1}\right)}\left(c_{3}\right)$. Hence, ηΨ¯1H1c3⊆Ψ¯2ηH1c3.
5. Now, let $z\in {\underline{\Psi }}_{2}^{\eta \left(H_{1}\right)}\left(c_{3}\right)$. Then, there exist unique *a* ∈ Θ_1_ such that *η*(*a*)*=z* and *η*(*a*)Ψ_2_(*c*_3_)⊆*η*(*H*_1_(*c*_1_′)). Let *x* ∈ *a*Ψ_1_(*c*_3_), that is, (*a*,  *x*) ∈ Ψ_1_(*c*_3_). Then, (*η*(*a*), *η*(*x*)) ∈ Ψ_2_(*c*_3_). Then, *η*(*x*) ∈ *η*(*a*)Ψ_2_(*c*_3_)⊆*η*(*H*_1_(*c*_1_′)). So, *η*(*x*) ∈ *η*(*H*_1_(*c*_1_′)). This implies that *x* ∈ *H*_1_(*c*_1_′). So, *a*Ψ_1_(*c*_3_)⊆*H*_1_(*c*_1_′). Then, a∈Ψ¯1H1c3. So, z=ηa∈ηΨ¯1H1c3. Hence, ${\underline{\Psi }}_{2}^{\eta \left(H_{1}\right)}\left(c_{3}\right)\subseteq$ηΨ¯1H1c3. Consequently, ηΨ¯1H1c3=${\underline{\Psi }}_{2}^{\eta \left(H_{1}\right)}\left(c_{3}\right)$.
6. Let x∈Ψ¯1H1c3 for any *c*_3_ ∈ *C*_3_. Then, ηx∈ηΨ¯1H1c3. Conversely, suppose that ηx∈ηΨ¯1H1c3. As *η* is bijection so x∈Ψ¯1H1c3. Similarly, we can show that ηx∈ηΨ¯1H1c3⇔x∈Ψ¯1H1c3.

**Remark 4 .**

With a similar technique, Lemma 1 can be proved but for the foresets.

**Theorem 17 .**

Let (*H*, *C*_1_) be soft weak isomorphic to (*F*, *C*_2_) with SWQI (*η*, *ζ*). Let (Ψ_2_, *C*_3_) be a SCPR over Θ_2_ and (*H*_1_, *C*_1_′)⊆(*H*, *C*_1_). Define Ψ_1_(*c*_3_)*=*{}(*x*,  *y*)∈Θ_1_ × Θ_1_:(*η*(*x*), *η*(*y*))∈Ψ_2_(*c*_3_){} for any *c*_3_ ∈ *C*_3_. Then, the following holds:

1. Ψ¯1H1c3 is an ideal of Θ_1_ iff ${\bar{\Psi }}_{2}^{\eta \left(H_{1}\right)}\left(c_{3}\right)$ is an ideal of Θ_2_ for all *c*_3_ ∈ *C*_3_
2. Ψ¯1H1c3 is a subquantale of Θ_1_ iff ${\bar{\Psi }}_{2}^{\eta \left(H_{1}\right)}\left(c_{3}\right)$ is a subquantale of Θ_2_ for all *c*_3_ ∈ *C*_3_
3. Ψ¯1H1c3 is a prime ideal of Θ_1_ iff ${\bar{\Psi }}_{2}^{\eta \left(H_{1}\right)}\left(c_{3}\right)$ is a prime ideal of Θ_2_ for all *c*_3_ ∈ *C*_3_
4. Ψ¯1H1c3 is a semiprime ideal of Θ_1_ iff ${\bar{\Psi }}_{2}^{\eta \left(H_{1}\right)}\left(c_{3}\right)$ is a semiprime ideal of Θ_2_ for all *c*_3_ ∈ *C*_3_
5. Ψ¯1H1c3 is a primary ideal of Θ_1_ iff ${\bar{\Psi }}_{2}^{\eta \left(H_{1}\right)}\left(c_{3}\right)$ is a primary ideal of Θ_2_ for all *c*_3_ ∈ *C*_3_

**Proof**

1. Let Ψ¯1H1c3 be an ideal of Θ_1_ for any *c*_3_ ∈ *C*_3_. We will show that ${\bar{\Psi }}_{2}^{\eta \left(H_{1}\right)}\left(c_{3}\right)$ is an ideal of Θ_2_. By Lemma 1 (3), we have ηΨ¯1H1c3=Ψ¯2ηH1c3.

Let p, q∈ηΨ¯1H1c3. Then, there exist u,v∈Ψ¯1H1c3 such that *η*(*u*)*=p* and *η*(*v*)*=q*. Since Ψ¯1H1c3 is ideal and (*η*, *ζ*) is SWQI so *p*∨*q=η*(*u*)∨*η*(*v*)*=η*(*u*∨*v*)∈ηΨ¯1H1c3.

Now, let *p*, *q* ∈ Θ_2_ such that *p* ≤ *q* and q∈ηΨ¯1H1c3. Then, there exist *u* ∈ Θ_1_ and v∈Ψ¯1H1c3 such that *η*(*u*)*=p* and *η*(*v*)*=q*. So, *η*(*u*) ≤ *η*(*v*) implies *η*(*u*∨*v*)*=η*(*u*)∨ηv=ηv∈ηΨ¯1H1c3. This implies that u∨v=v∈Ψ¯1H1c3. This implies that *u* ≤ *v* and Ψ¯1H1c3 are ideal so u∈Ψ¯1H1c3. Thus, ηu=p∈ηΨ¯1H1c3.

Finally, let *p* ∈ Θ_2_ and q∈ηΨ¯1H1c3. Then, there exist *u* ∈ Θ_1_ and v∈Ψ¯1H1c3 such that *η*(*u*)*=p* and *η*(*v*)*=q*. Since Ψ¯1H1c3 ideal, u∘1v∈Ψ¯1H1c3. Thus, *η*(*u*∘_1_*v*)=ηu∘2ηv=p∘2q∈ηΨ¯1H1c3. Similarly, q∘2p∈ηΨ¯1H1c3. Hence, ${\bar{\Psi }}_{2}^{\eta \left(H_{1}\right)}\left(c_{3}\right)$ is ideal of Θ_2_.

Conversely, suppose that ${\bar{\Psi }}_{2}^{\eta \left(H_{1}\right)}\left(c_{3}\right)$ = ηΨ¯1H1c3 be an ideal of Θ_2_ for any *c*_3_ ∈ *C*_3_. We will show that Ψ¯1H1c3 is ideal of Θ_1_.

Let u, v∈Ψ¯1H1c3. Then, *η*(*u*), *η*(*v*)∈ηΨ¯1H1c3. Since ηΨ¯1H1c3 is ideal so ηu∨v=ηu∨ηv∈ηΨ¯1H1c3. Then, by Lemma 5.2(5), u∨v∈Ψ¯1H1c3.

Now, let *u*, *v* ∈ Θ_1_ such that *u* ≤ *v* and v∈Ψ¯1H1c3. Then, u∨v=v∈Ψ¯1H1c3. Thus, *η*(*u*∨*v*)*=η*(*u*)∨*η*(*v*)*=η*(*v*)∈ηΨ¯1H1c3. This implies that *η*(*u*) ≤ *η*(*v*). Since ηΨ¯1H1c3 is ideal ηu∈ηΨ¯1H1c3. Then, by Lemma 5.2(5), u∈Ψ¯1H1c3. Finally, let *u* ∈ Θ_1_ and v∈Ψ¯1H1c3. Then, *η*(*u*) ∈ Θ_2_ and ηv∈ηΨ¯1H1c3. Since ηΨ¯1H1c3 is ideal, ηu∘2ηv∈ηΨ¯1H1c3, that is, ηu∘1v∈ηΨ¯1H1c3. Thus, *u*∘_1_*v*∈Ψ¯1H1c3. In a similar way, we can show that v∘1u∈Ψ¯1H1c3. This completes the proof.

The proof of (2)–(5) is similar to the proof of (1).

**Remark 5 .**

Theorem 17with a similar technique can be proved but for the foresets.

With the same arguments, the next Theorem 18 can be achieved.

**Theorem 18 .**

Let (*H*, *C*_1_) be soft weak isomorphic to (*F*, *C*_2_) with SWQI (*η*, *ζ*). Let (Ψ_2_, *C*_3_) be a SCTR over Θ_2_ and (*H*_1_, *C*_1_′)⊆(*H*, *C*_1_). Define Ψ_1_(*c*_3_)*=*{}(*x*,  *y*)∈Θ_1_ × Θ_1_:()*η*(*x*), *η*()*y*∈Ψ_2_(*c*_3_){} for any *c*_3_ ∈ *C*_3_. Then, the following holds:

1. Ψ¯1H1c3 is an ideal of Θ_1_ iff ${\underline{\Psi }}_{2}^{\eta \left(H_{1}\right)}\left(c_{3}\right)$ is an ideal of Θ_2_ for all *c*_3_ ∈ *C*_3_
2. Ψ¯1H1c3 is a subquantale of Θ_1_ iff ${\underline{\Psi }}_{2}^{\eta \left(H_{1}\right)}\left(c_{3}\right)$ is a subquantale of Θ_2_ for all *c*_3_ ∈ *C*_3_
3. Ψ¯1H1c3 is a prime ideal of Θ_1_ iff ${\underline{\Psi }}_{2}^{\eta \left(H_{1}\right)}\left(c_{3}\right)$ is a prime ideal of Θ_2_ for all *c*_3_ ∈ *C*_3_
4. Ψ¯1H1c3 is a semiprime ideal of Θ_1_ iff ${\underline{\Psi }}_{2}^{\eta \left(H_{1}\right)}\left(c_{3}\right)$ is a semiprime ideal of Θ_2_ for all *c*_3_ ∈ *C*_3_
5. Ψ¯1H1c3 is a primary ideal of Θ_1_ iff ${\underline{\Psi }}_{2}^{\eta \left(H_{1}\right)}\left(c_{3}\right)$ is a primary ideal of Θ_2_ for all *c*_3_ ∈ *C*_3_

## 6. Comparison

Yang and Xu [7] introduced rough approximations in quantale which is a kind of partially ordered algebraic structure with an associative binary operation. The main idea of work in [7] is based on equivalence relation equipped with congruence relation in quantale. In fact, the generalization of Pawlak's space is discussed in [7]. Further approximation of fuzzy substructures of quantale in crisp atmospheric space was discussed in [4]. Sometimes, it is difficult to find out an equivalence relation and then congruence while finding rough substructures in quantale. To remove this hurdle, soft binary relations are utilized in this paper. Since suitable soft binary relations are easy to find out, it is an easy approach to apply soft rough properties to approach different characterizations of soft rough structures in quantale with the help of aftersets and foresets.

## 7. Conclusion

The new combined effect of an algebraic structure quantale with rough and soft sets is presented by using soft binary relation, in this paper. The soft substructures of quantales like soft subquantale and soft ideal are discussed. The approximation w.r.t aftersets and foresets of these substructures by SBR which is an extended notion of Pawlak's rough approximation space are presented. The more generalized version of approximation space implied from SBR over Θ_1_ × Θ_2_ is employed. This new relation over Θ_1_ × Θ_2_ enables us to use the concept of aftersets and foresets to express the lower and upper approximation. Important results regarding to the approximation of soft substructures of quantales under SBR with some essential algebraic conditions such as compatible and complete relations are introduced. To emphasize and make a clear understanding, soft compatible and soft complete relations are focused, and these are interpreted by aftersets and foresets. Particularly, in our work, soft compatible and soft complete relations play an important role. Crux of these results is that whenever we approximate a soft algebraic structure of quantale, corresponding upper and lower approximations, are again the same kind of soft algebraic structure. Furthermore, we presented the soft quantale homomorphism and established the relationship of soft homomorphic images with their approximation under SBR.

In future, one can use this work and generalize it to different soft algebraic structures such as soft quantale modules, soft hypergroups, soft hyperquantales, and soft hyperrings. One can take motivation from our generalized approximation space and define new approximation spaces.

## Figures and Tables

**Table 1:** Binary operation subject to Θ

∘_1_	0	*p*	*q*	*r*	1
0	0	*p*	*q*	*r*	1
*p*	0	*p*	*q*	*r*	1
*q*	0	*p*	*q*	*r*	1
*r*	0	*p*	*q*	*r*	1
1	0	*p*	*q*	*r*	1

**Table 2:** Binary operation subject to Θ_1_.

O_1_	0	*p*	*q*	1
0	0	0	0	0
*p*	0	*p*	0	*p*
*q*	0	0	*q*	*q*
1	0	*p*	*q*	1

**Table 3:** Binary operation subject to Θ_2_.

O_2_	0′	*s*′	*p*′	*q*′	*r*′	1′
0′	0′	*s*′	*p*′	*q*′	*r*′	1′
*s*′	0′	*s*′	*p*′	*q*′	*r*′	1′
*p*′	0′	*s*′	*p*′	*q*′	*r*′	1′
*q*′	0′	*s*′	*p*′	*q*′	*r*′	1′
*r*′	0′	*s*′	*p*′	*q*′	*r*′	1′
1′	0′	*s*′	*p*′	*q*′	*r*′	1′

## Data Availability

No data were used to support this study.
